# New Insights Into Microbial Induced Calcium Carbonate Precipitation Using *Saccharomyces cerevisiae*

**DOI:** 10.3389/fmicb.2022.904095

**Published:** 2022-04-29

**Authors:** Zhimin Li, Tianxiao Li

**Affiliations:** ^1^Joint International Research Laboratory of Environmental and Social Archaeology, Shandong University, Qingdao, China; ^2^Institute of Cultural Heritage, Shandong University, Qingdao, China

**Keywords:** *Saccharomyces cerevisiae*, biomineralization, microbially induced calcium carbonate precipitation, transcriptome analysis (RNAseq), culture condition screening

## Abstract

*Saccharomyces cerevisiae* plays an important role in the mineralization of many metal ions, but it is unclear whether this fungus is involved in the mineralization of calcium carbonate. In this study, *S. cerevisiae* was cultured under various conditions to explore its ability to perform microbially induced calcium carbonate precipitation (MICP). Organic acids, yeast extract, and low-carbon conditions were the factors influencing the biomineralization of calcium carbonate caused by *S. cerevisiae*, and biomolecules secreted by the fungus under different conditions could change the morphology, size, and crystal form of the biosynthesized mineral. In addition, transcriptome analysis showed that the oxidation of organic acids enhanced the respiration process of yeast. This implied that *S. cerevisiae* played a role in the formation of calcium carbonate through the mechanism of creating an alkaline environment by the respiratory metabolism of organic acids, which could provide sufficient dissolved inorganic carbon for calcium carbonate formation. These results provide new insights into the role of *S. cerevisiae* in biomineralization and extend the potential applications of this fungus in the future.

## Introduction

*Saccharomyces cerevisiae* (baker’s yeast) is a common unicellular specie that is widely used in food applications (Krumov and Posten, 2011). Owing to the ability of yeast to accumulate metal ions from aqueous solutions to produce minerals, *S. cerevisiae* is also widely used in biomineralization studies (Krumov et al., 2009; Krumov and Posten, 2011; Durán and Favaro, 2018; Qin et al., 2020; Geetha et al., 2021).

*Saccharomyces cerevisiae* acts as both a template and biocarbon source in biomineralization. Some materials, such as three-dimensional porous foam-like Na_3_V_2_(PO_4_)_3_@C composites, mesoporous LiFePO_4_, and carbon-coated LiFePO_4_, have been synthesized based on yeast cells (Zhang et al., 2013; Cao et al., 2017; Hou et al., 2018a,b; Chen et al., 2019a,b; An et al., 2021), providing a promising method to prepare high-performance materials for batteries. As a template, S. *cerevisiae* is also involved in the biosynthesis of other mesoporous materials, such as SiO_2_, TiO_2_, and Li_2_O-MgO-P_2_O_5_-TiO_2_ nanocrystalline glass (He et al., 2011a; Du et al., 2013; Cui and Liu, 2017), and a series of metal-phosphate-protein nanocomposites can be synthesized with yeast cells (He et al., 2011b). In addition, research has shown that the crystallization of calcium phosphate and calcium carbonate can be controlled when the yeast cells are modified with a layer-by-layer (LBL) self-assembly process (Wang et al., 2008; Huang and Wang, 2012; Huang et al., 2014; Wei et al., 2020).

The metabolic activity of *S. cerevisiae* also contributes to the formation of minerals. Previous studies have reported that *S. cerevisiae* may play an important role in the bioremediation of heavy metals (Krumov et al., 2009; Qin et al., 2020; Geetha et al., 2021). It can absorb Cd^2+^ and Pb^2+^ in industrial wastewater and synthesize CdS and PbS nanocrystals intracellularly with the participation of H_2_S (Yu et al., 1996; Meenal et al., 2003; Krumov et al., 2009; Krumov and Posten, 2011; Qin et al., 2020), which is produced by the sulfate assimilation pathway under fermentation conditions (Sun et al., 2020). Sun et al. (2020) modified the relevant genes in this pathway, and the modified yeast cells promoted the removal of Hg, Pb, and Cu from polluted water. Heavy metals can also be precipitated extracellularly as phosphate by the phosphatase action of *S. cerevisiae* (He et al., 2009; Krumov and Posten, 2011; Liang et al., 2016a,b; Nie et al., 2017; Gerber et al., 2018; Jiang et al., 2018; Shen et al., 2018; Zheng et al., 2018; Kolhe et al., 2020; Qin et al., 2020; Geetha et al., 2021). Some studies have focused on the formation of uranium phosphate biomineral with yeast to treat uranium waste, and *S. cerevisiae* is considered to have great potential in the bioremediation of uranium-contaminated areas (Liang et al., 2016b; Nie et al., 2017; Gerber et al., 2018; Shen et al., 2018; Zheng et al., 2018; Kolhe et al., 2020). Rare earth elements, such as Ce, Yb, and Sm, can also be precipitated extracellularly by yeast through phosphate mineralization (Jiang et al., 2010, 2018, 2012). In addition, endogenous CO_2_ produced by the respiration of yeast can interact with exogenous metal ions under alkaline conditions to form carbonates intracellularly. This method has been used to synthesize some insoluble carbonate nanomaterials, including BaCO_3_ and CaCO_3_ (Ma et al., 2011; Chang et al., 2021).

CaCO_3_ produced by microorganisms is common in nature and can be observed in numerous microorganisms with metabolic capabilities such as photosynthesis, iron reduction, sulfate reduction, ammonification, nitrate reduction (denitrification), urea hydrolysis, and the oxidation of organic acids (Li et al., 2018; Qin et al., 2020; Justo-Reinoso et al., 2021; Lin et al., 2021). Microbially induced calcium carbonate precipitation (MICP) is an emerging technique that has been used to address soil amelioration, building material rehabilitation, the conservation of stone monuments, and environmental issues (Reeksting et al., 2020; Lin et al., 2021). To date, most studies on MICP have focused on ureolytic bacteria because of the higher and faster precipitation rate of CaCO_3_ by the bacteria compared to other types of microorganisms (Reeksting et al., 2020; Justo-Reinoso et al., 2021; Lin et al., 2021). However, some drawbacks of ureolytic bacteria limit its application. For example, the fast rate of CaCO_3_ precipitation leads to small particle size and non-uniform distribution (Justo-Reinoso et al., 2021), and the production of ammonia and nitrogen oxide can contribute to environmental concerns (Reeksting et al., 2020; Justo-Reinoso et al., 2021; Lin et al., 2021). In recent years, some aerobic non-ureolytic bacteria and denitrifying bacteria with significant environmental advantages have become another promising alternative to MICP.

*Saccharomyces cerevisiae* is a facultative anaerobic fungus that is widely used in brewing and in the production of food. The large amounts of CO_2_ produced by the respiration of *S. cerevisiae* can provide dissolved inorganic carbon for CaCO_3_ precipitation (Ma et al., 2011). In addition, advantages such as low cost, a lack of toxicity, no pollution, a wide pH tolerance, and providing the nucleating agent suggest that *S. cerevisiae* has great potential in MICP (He et al., 2011a; Zhang et al., 2013). However, in previous studies, the formation of CaCO_3_ produced by this fungus was artificially controlled; for example, through the addition of alkaline reagents or LBL modification (Wang et al., 2008; Ma et al., 2011; Chang et al., 2021). Whether *S. cerevisiae* has a natural capacity for MICP is not clear.

In the present study, we determined the role of S. *cerevisiae* in MICP. Different media were used to culture the fungus to determine which factors were related to the process of *S. cerevisiae* induced calcium carbonate precipitation. These findings will provide new insights into the biomineralization function of *S. cerevisiae*, and a new option for the application of MICP.

## Materials and Methods

### Cultivation of *Saccharomyces cerevisiae* Under Various Conditions

In the present study, *S. cerevisiae* was purchased from Angel Yeast Co., Ltd., (China). The dried yeast was dissolved in water and incubated with the medium of potato dextrose agar at 30°C to obtain pure strains. The isolated yeast was cultured with seven different liquid media based on B4 medium (4 g yeast extract, and 10 g glucose in 1 L water) and Czapek-Dox medium (3 g NaNO_3_, 0.5 g KCl, 1 g K_2_HPO_4_, 0.5 g MgSO_4_⋅7H_2_O, 0.01 g FeSO_4_, and 20 g sucrose in 1 L water; Li et al., 2018). The detailed compositions of the seven media are shown in Table 1 (groups 1–7). The concentration of Ca^2+^ in each medium was adjusted to 0.64 g/L, and urea was added until it reached a concentration of 0.95 g/L. All chemicals used in the study are of analytical grade.

**TABLE 1:** The composition of media under different conditions.

Groups	Medium	Calcium sources	Other additives
1	B4	Calcium acetate	–
2	B4	Calcium lactate	–
3	B4	Calcium citrate	–
4	B4	CaCl_2_	–
5	B4	CaCl_2_	Urea
6	B4	CaSO_4_	–
7	Czapek-Dox	Calcium acetate	–
8	B4	Calcium acetate	Without glucose
9	B4	Calcium lactate	Without glucose
10	B4	CaCl_2_	Without glucose
11	–	Calcium acetate	Tryptone, glucose
12	–	Calcium lactate	Tryptone, glucose
13	–	Calcium acetate	Tryptone
14	–	Calcium lactate	tryptone

*The concentrations of Ca^2+^ and urea were 0.64 and 0.95 g/L, respectively.*

Three parallel samples were performed for each medium in this study. *S. cerevisiae* was adjusted to 1 × 10^5^ CFU/ml with 100 ml medium and shaken at 150 r/min. It was incubated at 30°C for 4 days, and then the biomasses were analyzed to detect the formation of crystals.

Calcium carbonates were only found in the biomasses from two organic media with calcium acetate and calcium lactate (group 1 and group 2, respectively). Therefore, in the subsequent study, only the two media (group 1, group 2) were used, besides, group 4, which has the same media with group 1 and group 2, was selected as control. *S. cerevisiae* was incubated with the three media at 30°C for 12 days. Five milliliters of media were extracted before culturing and at the first, third, fifth, seventh and twelfth day. The samples were then filtered through 0.22-μm membranes, and the sterilized media were stored at 4°C. In addition, biomasses on the twelfth day were sterilized to prevent contamination of the environment with live yeast and dried to constant weight at 30°C. After being ground, powder samples were stored at room temperature for further analysis.

### Cultivation of *Saccharomyces cerevisiae* Under Low-Carbon Conditions

According to the transcriptome results, we moderated the composition of the medium used for *S. cerevisiae* culture. Glucose was removed from the media and organic acids became the main source of carbon, thus creating a low-carbon condition for yeast growth (Table 1, groups 8–10). There are some carbon sources in yeast extract, so a new nitrogen source, tryptone, was added in the medium. Theses normal and low-carbon conditions were shown in Table 1, group 11–14, and the concentration of tryptone was 10 g/L. The process of culturing *S. cerevisiae* was the same as that described above.

No obvious calcium carbonate was found in the media with tryptone, and further analysis of the liquid media and biomasses was only performed for groups 8–10.

### Characterization of the Metabolic Activity of *Saccharomyces cerevisiae*

The pH value and the concentration of calcium and organic acid in the liquid media were measured. The pH value of the samples was detected by an S210-K pH meter (Mettler Toledo, Switzerland). The concentration of calcium in the samples was analyzed with an inductively-coupled plasma optical emission spectrometer (ICP-OES, Agilent 5100, United States) based on the Chinese standard (JY-T 0567-2020, 2020). The operating parameters for calcium are listed in Supplementary Table 1. Changes in the acetate and lactate in group 1 and group 2, respectively, were determined with high-performance liquid chromatography (HPLC, Agilent 1260, United States). The column temperature was 35°C and potassium dihydrogen phosphate was selected as the mobile phase, for which the pH was adjusted to 2.55. Samples were detected at the wavelengths of 210 nm with a flow rate of 0.5 ml/min.

### Characterization of *Saccharomyces cerevisiae*-Induced Crystals

A polarizing microscope (DM4P, Leica, Germany) was used to determine whether crystals could be produced by S. *cerevisiae* in different conditions. The minerals produced were then analyzed using a scanning electron microscope (SEM, Quattro S, Thermo Fisher, United States) with an energy-dispersive spectroscopy (EDS, XFlash 6160, Bruker, Germany) probe. The powder samples were analyzed in low-vacuum mode using an accelerating voltage of 15 kV for SEM and EDS.

Minerals precipitated by *S. cerevisiae* were further detected using an X-ray polycrystal diffractometer (XRD, D8 Advance, Bruker, Germany). The samples were analyzed over the range 10–70° 2θ at a scan rate of 1°/min in 0.02° increments. Fourier transform infrared spectroscopy (FTIR, Nicolet iN 10, Thermo Fisher, United States) was also used to analyze the minerals. The samples were mixed with potassium bromide (KBr, IR grade), and they were analyzed in the 4000–400 cm^–1^ range (36 scans with a spectral resolution of 0.48 cm^–1^).

### Transcriptome Analysis

*Saccharomyces cerevisiae* cultured with different media (group 1, group 2, and group 4) for 2 days was selected for transcriptome sequencing. Library construction and RNA-sequencing were performed by GENEWIZ (Jiangsu, China). The total RNA of each sample was extracted using a MagZol Reagent Kit (Magen, Guangdong, China), and 1 μg total RNA was used for library preparation. The cDNA libraries were constructed according to the manufacturer’s protocol (Illumina, San Diego, California, United States). The separated mRNA was fragmented, and first- and second-strand cDNAs were synthesized and end-repaired. A fragment of about 400 bp in length was recovered and used as a template for polymerase chain reaction (PCR) amplification. The libraries were validated using an Agilent 2100 Bioanalyzer (Agilent Technologies, Palo Alto, CA, United States), and the PCR products were quantified by a Qubit3.0 Fluorometer (Invitrogen, Carlsbad, CA, United States). Then, the libraries were loaded on an Illumina NovaSeq instrument according to the manufacturer’s instructions (Illumina, San Diego, CA, United States) for sequencing. All samples were sequenced with three biological replicates.

After sequencing, technical sequences in the raw data, including adapters, PCR primers or fragments thereof, and bases with quality lower than 20, were removed, and they were then aligned to the reference strain *S. cerevisiae* S288C genome (GenBank assembly accession: GCA_000146045.2). Gene and isoform expression levels from the clean data were estimated with HTSeq (v0.6.1). Differential expression analysis was conducted using the DESeq2 Bioconductor package, and genes with P-adjust of genes <0.05 were considered to be differentially expressed genes (DEGs). The DEGs were then functionally annotated with Gene Ontology (GO) databases by GOSeq (v1.34.1). The enriched GO terms were categorized into three different functional groups, namely, biological process (BP), cellular component (CC), and molecular function (MF). Further, the DEGs were subjected to pathway analysis using the Kyoto Encyclopedia of Genes and Genomes (KEGG) database. Metabolic pathways of yeast related to the acetate and lactate were identified. Over-represented *P*-values and P-adjust values lower than 0.05 were considered as significant GO terms and KEGG pathways, respectively. Raw sequences were deposited in the NCBI Sequence Read Archive (SRA) database (Accession numbers: SAMN24662185-SAMN24662187, SAMN24662193-SAMN24662198).

## Results

### Factors Related to *Saccharomyces cerevisiae*-Induced Calcium Carbonate Precipitation

Fourteen different media were used to culture yeast cells (Table 1), and minerals were observed in most samples (Supplementary Figure 1). However, the XRD and FTIR results indicated that most of the minerals belonged to calcium phosphates (Supplementary Figures 2, 3), which was contributed by the phosphatase action of *S. cerevisiae* (He et al., 2009; Krumov and Posten, 2011; Liang et al., 2016a,b; Nie et al., 2017; Gerber et al., 2018; Jiang et al., 2018; Shen et al., 2018; Zheng et al., 2018; Kolhe et al., 2020; Qin et al., 2020; Geetha et al., 2021).

**FIGURE 1:**
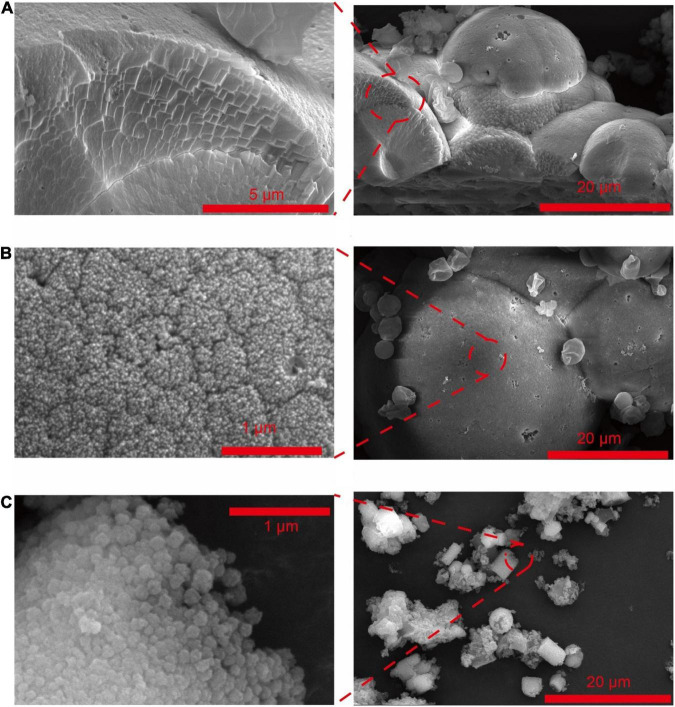
SEM of CaCO_3_ synthesized by *Saccharomyces cerevisiae* with different culturing conditions. **(A)** Acetate in B4 medium. **(B)** Lactate in B4 medium. **(C)** Acetate in B4 medium without glucose.

**FIGURE 2:**
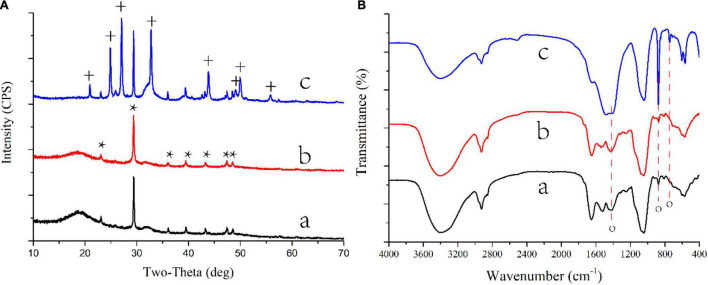
XRD patterns **(A)** and FTIR spectra **(B)** of CaCO_3_ on biomass synthesized by *S. cerevisiae* in different conditions. a, acetate in B4 medium; b, lactate in B4 medium; c, acetate in B4 medium without glucose; *, the base peak of calcite; +, the peak of vaterite. o, the base peak of calcium carbonate.

Five calcium sources, including calcium acetate, calcium lactate, calcium citrate, calcium chloride, and calcium sulfate, were used to evaluate the biomineralization ability of *S. cerevisiae* (Table 1 groups 1–4, 6). CaCO_3_ could only crystallize with the presence of calcium acetate and calcium lactate (Figures 1A,B, 2A,B and Supplementary Figures 1A1,B1), which suggested that organic acid content related to crystallization of calcium carbonate. Calcium citrate and calcium sulfate were insoluble, and we did not find any crystal among yeast cells cultured with the two salts. It implied that yeast could not use insoluble calcium salts to synthesis mineral. These results suggested that soluble organic acid calcium salt related to the biomineralization of CaCO_3_.

Different nitrogen sources, including urea, nitrate, yeast extract, and peptone, were also used to culture *S. cerevisiae* (Table 1 groups 1, 5, 7, 11–14). Crystallized CaCO_3_ was formed only when the yeast extract was the nitrogen source (Figures 1A,B, 2A,B, Supplementary Figures 1A1,B1). These results indicated that yeast extract was a factor contributing to the formation of calcium carbonated induced by *S. cerevisiae*.

The composition of carbon sources could also affect *S. cerevisiae* induced CaCO_3_ precipitation (Table 1 groups 1–3, 8–14). When yeast was cultured in a low-carbon medium from which glucose was removed, CaCO_3_ was only crystallized with the presence of calcium acetate (Figures 1C, 2c and Supplementary Figure 1F1). In addition, the metabolism of glucose could also influence the crystal forms and morphology of calcium carbonate. The crystals of CaCO_3_ were spherical calcite with the existence of glucose (Figures 1A, 2A), while after the removal of glucose, vaterite, an unstable crystal form of CaCO_3_, were also observed (Figure 2C) and the crystals were cylindrical shape (Figure 1C).

### Metabolic Characterization of *Saccharomyces cerevisiae* During Biomineralization

**FIGURE 3:**
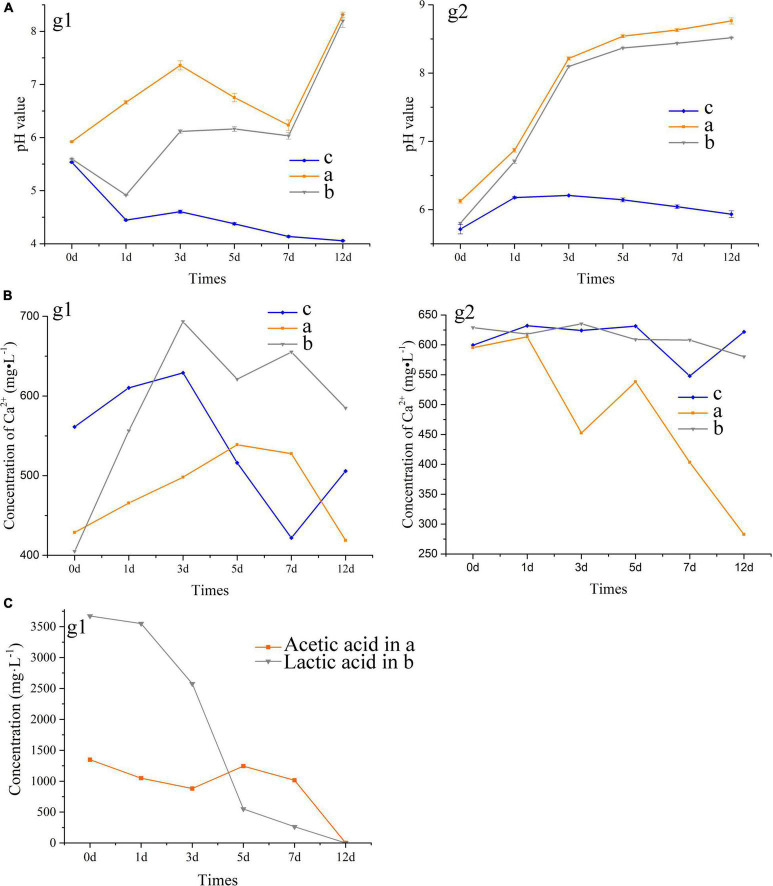
Tendency of pH value **(A)** and the concentration of Ca^2+^ **(B)** and organic acids **(C)** in the extracellular metabolites of *S. cerevisiae*. a, acetate in B4 medium; b, lactate in B4 medium; c, acetate in B4 medium without glucose. g1, the medium with glucose; g2, the medium without glucose.

During 12-day cultures of *S. cerevisiae* with different media, only the media with acetate and lactate transformed from acid to alkaline, and the changes of pH in the two media showed three inflection points on the first, third, and seventh days (Figure 3A). However, the transition of the media to alkaline only occurred after 7 days of incubation. The change of acetate in the medium was similar with to the change in pH, with both showing a sharp change beginning after the seventh day and an extreme value appearing on the third day (Figure 3C), suggesting a relationship between the pH increase and the oxidation of acetate. However, there was less of a relationship between the change of pH and that of lactate in the medium. Lactate decreased continuously during incubation, with an especially sharp decrease from 2,577 to 549 mg/L occurring between the third and fifth days (Figure 3C), while the pH of the medium remained relatively stable on these days. As shown in Figure 3a2, the concentration of calcium dissolved in the different media varied greatly after autoclaving, although the initial concentration of calcium was same. Reaction and precipitation among the contents in the media during autoclaving may have been responsible for this result. With the growth of *S. cerevisiae*, the concentration of calcium showed a trend of first increasing and then decreasing, where the calcium ions dissolved in the media reached the maximum on the third or fifth day (Figure 3A). The changes in the dissolved calcium ions in the media showed less of a relationship with the changes in the pH and organic acids. In addition, the final concentration of calcium in the media containing calcium chloride was lower than that in the media containing calcium lactate, which suggested that the addition of lactate decreased the precipitation rate of calcium, although lactate could induce the formation of calcium carbonate and calcium phosphate.

After glucose was removed from the media, we found that the initial pH and the concentration of soluble calcium ion increased, which indicated that glucose promoted the decrease of pH and the precipitation of calcium in the medium (Figure 3B). The pH in the media with acetate or lactate continued to increase with the growth of *S. cerevisiae*, and a sharp shift from acid to alkaline appeared in the first 3 days. In addition, the removal of glucose promoted the decrease of calcium in the media containing calcium acetate, from 595 to 282 mg/L (Figure 3B). However, with or without glucose, the final concentration of calcium ions in the media containing calcium lactate was consistent at about 580 mg/L. These results suggested that acetate as the main carbon source would promote the precipitation of CaCO_3_ induced by *S. cerevisiae*.

### Overview of the Differentially Expressed Genes

The third day was a major inflection point for metabolic activity during the growth of *S. cerevisiae*. Nine samples cultured for 2 days with three media were selected for transcriptome analysis to understand the effects of different calcium sources on yeast growth. The results of Pearson correlation coefficient and principal component analysis showed that there was an outlier in each group (Supplementary Figure 4), and two close samples in each group were used for further transcriptome analysis.

There were 1426 DEGs (P-adjust < 0.05, log_2_ fold change ≥ 1) in the sample C-Y (comparison of transcripts from the yeast cultured under between calcium acetate medium and control), containing 785 genes showing upregulation and 641 genes showing downregulation. A higher number of DEGs was found in C-R (comparison of transcripts from the yeast cultured under between calcium lactate medium and control), with 915 significantly upregulated genes and 877 downregulated genes. We also compared the transcripts between calcium acetate medium and calcium lactate medium, where the genomes from calcium acetate medium were used as a reference (Y-R), and most of the DEGs belonged to downregulated genes. In addition, the order of the numbers of unique DEGs was similar with that of the total DEGs in each group, with 496, 275, and 68 unique DEGs in C-R, C-Y, and Y-R, respectively.

### Gene Ontology Terms and Kyoto Encyclopedia of Genes and Genomes Pathway Enrichment Analysis

**FIGURE 4:**
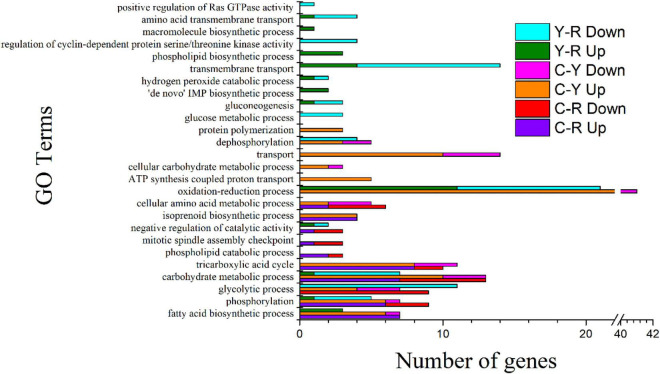
GO Terms of biological process identified in *S. cerevisiae* transcripts in response to different conditions. C-R, comparison of transcripts from the yeast cultured under between calcium acetate medium and control; C-Y, comparison of transcripts from the yeast cultured under between calcium lactate medium and control Y-R, comparison of transcripts from the yeast cultured under between calcium acetate medium and calcium lactate medium; Up, up-regulated genes; Down, down-regulated genes.

The enriched GO terms of the samples are shown in Figure 4 and Supplementary Figure 5. When acetate or lactate was used for the culture of *S. cerevisiae*, the predominant GO terms in molecular function (MF) were ATP binding, the most active cellular component (CC) term was integral component of membrane, and in biological process (BP), most DEGs were involved in the process of energy metabolism, including carbohydrate metabolic process, fatty acid biosynthetic process, glycolytic process, and tricarboxylic acid cycle (Figure 4). These results suggested that the decrease of acetate and lactate mentioned above may have been related to the metabolic activities of *S. cerevisiae*, especially the energy metabolism. The enrichment analysis for the DEGs of Y-R revealed that 2, 17, and 17 GO terms were grouped into CC, MF, and BP, respectively. Most DEGs in the GO terms were downregulated, especially in the GO terms of glycolytic process, glucose metabolic process, and carbohydrate metabolic process, in which all the DEGs were downregulated. This suggested that acetate was more capable of promoting the energy metabolic activity of yeast compared with lactate.

**FIGURE 5:**
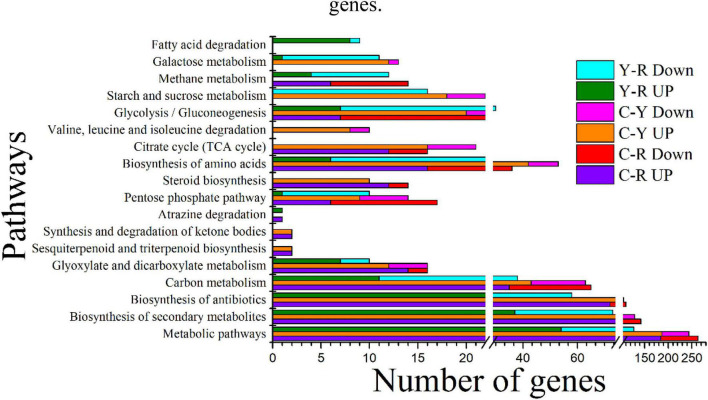
Top 15 significant KEGG pathways identified in *S. cerevisiae* transcripts in response to different conditions. C-R, comparison of transcripts from the yeast cultured under between calcium acetate medium and control; C-Y, comparison of transcripts from the yeast cultured under between calcium lactate medium and control Y-R, comparison of transcripts from the yeast cultured under between calcium acetate medium and calcium lactate medium; Up, up-regulated genes; Down, down-regulated genes.

KEGG enrichment was conducted to identify the differences in major biological pathways of *S. cerevisiae* cultured with different media. Most of the DEGs were mapped into metabolic pathways. The top 15 significant pathways are shown in Figure 5. A large proportion of DEGs took part in the pathways, metabolic pathways, biosynthesis of secondary metabolites, biosynthesis of antibiotics, carbon metabolism, and biosynthesis of amino acids when acetate and lactate were added, and the number of the mapped upregulated genes was greater than that of the mapped downregulated gens. This was consistent with the results of GO terms analysis. In addition, we found that the citrate cycle (TCA cycle) was also an enriched pathway of sample C-Y, and most of the DEGs in this pathway were upregulated, which suggested a relationship between the reduction of acetate and the carbon dioxide production. After comparing the transcripts of the sample Y with the sample R, most of the DEGs in the sample R were also mapped the main pathways mentioned above. Interestingly, all the pathways mentioned above, except for glyoxylate and dicarboxylate metabolism, included more downregulated genes than upregulated ones, indicating that lactate was less capable of promoting substance and energy metabolism. These results may provide an explanation for the finding that the reduction of calcium in the medium with acetate was higher than that in the medium with lactate.

## Discussion

In this study, we determined the ability of *S. cerevisiae* in regard to MICP without artificial modification, and found that soluble organic acid and yeast extract could promote the extracellular formation of CaCO_3_. In addition, low-carbon conditions with the use of organic acid as the main carbon source increased the precipitation rate of CaCO_3_, but CaCO_3_ was only observed with the presence of acetate in the medium. These findings provide some new insights into the carbon cycle, and a new option for the application of MICP.

Creating an alkaline environment is the primary role of microorganisms in MICP (Li et al., 2018; Qin et al., 2020; Reeksting et al., 2020; Justo-Reinoso et al., 2021; Lin et al., 2021). Many metabolic activities, such as photosynthesis, sulfate reduction, urea hydrolysis, and the oxidation of organic acids, can contribute to an increase of the pH surrounding microbial cells (Li et al., 2018; Qin et al., 2020; Reeksting et al., 2020; Justo-Reinoso et al., 2021; Lin et al., 2021). In the present study, sulfate, urea, and organic acids including acetate, lactate, and citrate were used to culture *S. cerevisiae*. The increase of pH was only observed with the presence of acetate and lactate. During the first 3 days of incubation, the pH increased, accompanied by a decrease in added organic acids. Additionally, some DEGs were enriched in the glyoxylate and dicarboxylate metabolism pathway, in which lactate and acetate were oxidized for energy metabolism, and both of these organic acids contributed to the up-regulation of DEGs in the TCA cycle, a primary process of aerobic respiration. This finding suggested that the aerobic respiration of *S. cerevisiae* contributed to the oxidation of these organic acids, which led to the increase of pH and dissolved inorganic carbon, as shown in Equations (1)–(3) (Dhami et al., 2014).


(1) $$\text{R}-{\text{COO}}^{-}+2\,{\text{O}}_{2}\to {\text{CO}}_{2}+{\text{H}}_{2}\,\text{O}+{\text{OH}}^{-}$$

(2) $$2\,{\text{CO}}_{2}+{\text{OH}}^{-}\to {\text{CO}}_{2}+{\text{HCO}}_{3}^{-}$$

(3) $$2\,{\text{HCO}}_{3}^{-}+{\text{Ca}}_{2}^{+}\to \mathrm{CaCO3}+{\text{CO}}_{2}+{\text{H}}_{2}\,\text{O}$$

Citrate, a major intermediate product in the TCA cycle, was also used to culture *S. cerevisiae*, but there was no CaCO_3_ detected in the biomass. The added calcium citrate was insoluble, and few citrates could be absorbed and oxidized by the yeast. This may have been the reason why the mineral was not found around the yeast cells. Other organic acids involved in the TCA cycle should be evaluated in future studies to compare their effects on yeast-induced calcium carbonate precipitation.

The nucleation site is considered another key factor for calcium carbonate precipitation, and the cell surfaces of bacteria are negatively charged, which can induce Ca^2+^ to accumulate on their surfaces and act as a nucleation site for CaCO_3_ (Dhami et al., 2014, 2013; Li et al., 2018; Qin et al., 2020; Reeksting et al., 2020; Intarasoontron et al., 2021; Justo-Reinoso et al., 2021). However, in the present study, the precipitated calcium carbonates were not distributed along the surface of yeast cells. A previous study reported that the cell wall of yeast was mainly composed of weakly electronegative polysaccharides (Cabib et al., 2001), which was the reason that Ca^2+^ could not accumulate on the surface of yeast. This suggests that yeast cells cannot provide a nucleation site for CaCO_3_ precipitation. This may have been a major reason why yeast cells were first modified by LBL before they were used to prepare CaCO_3_ in previous studies (Wang et al., 2008; Ma et al., 2011; Chang et al., 2021).

It is known that glucose can inhibit the metabolism of other carbon sources when multiple carbon sources are used to culture microorganisms (Makuc et al., 2001), which is called the glucose effect. This was supported by the findings of the present study that the concentration of acetate in the medium containing glucose remained relatively constant until the glucose was consumed and then decreased rapidly, accompanied by a rapid increase of pH. After the removal of glucose, the pH and the precipitation rate of calcium ions increased, which suggested that glucose could inhibit the ability of yeast in regard to MICP. Interestingly, although the removal of glucose prompted the rise of pH in the media with lactate, CaCO_3_ could not be found around yeast cells. Sufficient Ca^2+^ and CO_3_^2–^ are the key factors that govern the process of calcium carbonate precipitation (Dhami et al., 2013). Sufficient CO_3_^2–^ was provided by the reaction between CO_2_ produced by yeast respiration and the added alkaline solution in previous studies (Ma et al., 2011; Chang et al., 2021). The oxidation of lactate transformed the media from acid to alkaline, which indicated that CO_2_ produced by *S. cerevisiae* through the metabolism of lactate alone might not lead to the formation of enough CO_3_^2–^. In addition, CaCO_3_ was only found in the media with yeast extract as a nitrogen source in the present study. This suggested that the metabolism of different nitrogen sources by *S. cerevisiae* could also limit the production of CO_3_^2–^.

Vaterite is a metastable crystal form of calcium carbonate that finally transforms into the stable form calcite. Our previous study reported that the metabolites of acetate secreted by fungus could control the transformation between calcite and vaterite (Li et al., 2018). This result was confirmed by the present study, but it was only observed under low-carbon conditions. In addition, the morphology and size of calcium carbonate are also influenced by the metabolites of acetate under different conditions. Chang et al. (2020) found that biomolecules secreted by yeast could transform calcium carbonate from amorphous to calcite and from nanoparticles to mesoporous nano/microspheres. This finding suggested that the metabolites of acetate produced by *S. cerevisiae* under different conditions might influence the morphology, size, and crystal forms of the biosynthesized calcium carbonate.

## Conclusion

The role of *S. cerevisiae* in MICP was clarified in the present study, in which *S. cerevisiae* created an alkaline environment through the respiratory metabolism of organic acids and provided sufficient dissolved inorganic carbon for CaCO_3_ formation. The biosynthesized CaCO_3_ could be influenced by the types of organic acid, glucose, and yeast extract in terms of precipitation rate, morphology, size, and crystal forms. These findings provide some new insights into the role of *S. cerevisiae* in biomineralization. The advantages of this fungus such as its low cost, environmental friendliness, and ease of modification suggest that it may be new option for the application of MICP in the future.

## Data Availability Statement

The datasets presented in this study can be found in online repositories. The names of the repository/repositories and accession number(s) can be found in the article/Supplementary Material.

## Author Contributions

TL contributed to conception and design of the study, performed the statistical analysis, and wrote the first draft of the manuscript. ZL wrote sections of the manuscript and contributed to manuscript revision. Both authors contributed to the article and approved the submitted version.

## Conflict of Interest

The authors declare that the research was conducted in the absence of any commercial or financial relationships that could be construed as a potential conflict of interest.

## Publisher’s Note

All claims expressed in this article are solely those of the authors and do not necessarily represent those of their affiliated organizations, or those of the publisher, the editors and the reviewers. Any product that may be evaluated in this article, or claim that may be made by its manufacturer, is not guaranteed or endorsed by the publisher.
