# Supplementary material for: New Insights Into Microbial Induced Calcium Carbonate Precipitation Using Saccharomyces cerevisiae
Source: Front Microbiol. 2022 Apr 29;13:904095. doi: 10.3389/fmicb.2022.904095 (PMC9100588; doi:10.3389/fmicb.2022.904095)
Supplement: Supplementary file 1 [file Data_Sheet_1.docx]

**New insights into microbial induced calcium carbonate precipitation using *Saccharomyces cerevisiae***

Tianxiao Li^1, 2*^

1Joint International Research Laboratory of Environmental and Social Archaeology, Shandong University, Qingdao, Shandong, 266237, China

2Institute of Cultural Heritage, Shandong University, Qingdao, Shandong, 266237, China

*Corresponding Author: Tianxiao Li, Email: li_tianxiao@sdu.edu.cn

**Supplement Contents**

This supplement contains, in the following order:

Table S1. ICP-OES operating conditions for the analysis of calcium in the media.

Figure S1. EDS analysis of the precipitated crystals.

Figure S2. FTIR of minerals synthesized by S. cerevisiae in different conditions.

Figure S3. XRD of crystals on biomass synthesized by S. cerevisiae in different conditions.

Figure S4. Pearson correlation heatmap between different samples.

Figure S5. GO Terms of molecular function (a) and cellular components (b) identified in S. *cerevisiae* transcripts in response to different conditions.

| Table S1. ICP-OES operating conditions for the analysis of calcium in the media. | |
| --- | --- |
| Operating conditions | |
| Wavelengths | 317.933 nm |
| Plasma position | Radial |
| Power | 1.2 kW |
| Nebulizer gas | 0.7 L/min |
| Plasma gas | 12 L/min |
| Auxiliary gas | 1 L/min |
| Read time | 3 × 5 s |
| Observation height | 8 mm |


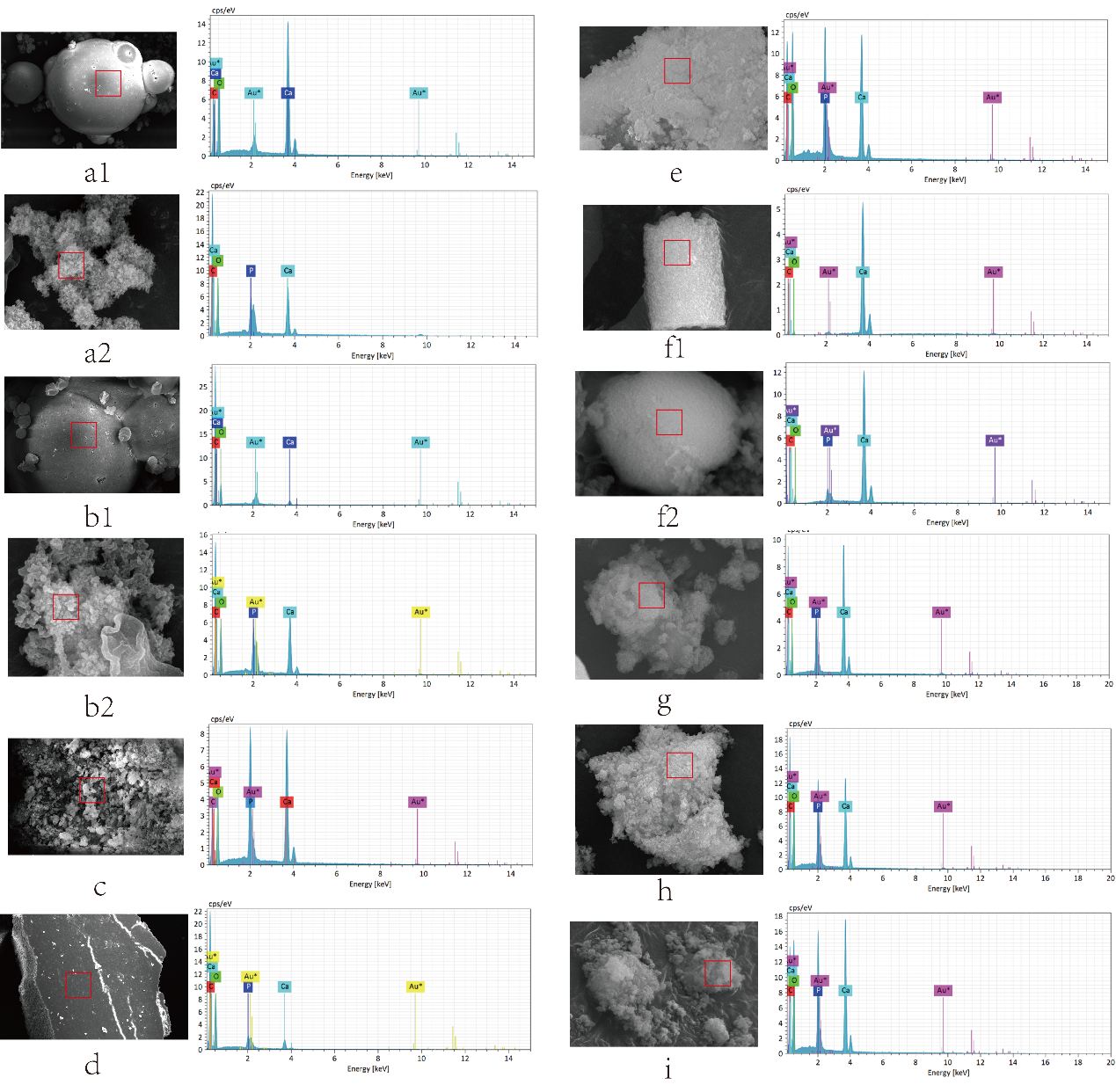


Figure S1. EDS analysis of the precipitated crystals. Group1, acetate in B4 medium; Group2, lactate in B4 medium; Group4, CaCl_2_ in B4 medium; Group5, CaCl_2_ in B4 medium with urea; Group7, acetate in Czapek-Dox medium; Group8, acetate in B4 medium without glucose; Group9, lactate in B4 medium without glucose; Group10, CaCl_2_ in B4 medium without glucose; Group13, acetate in the medium of tryptone; the red box was the testing site.


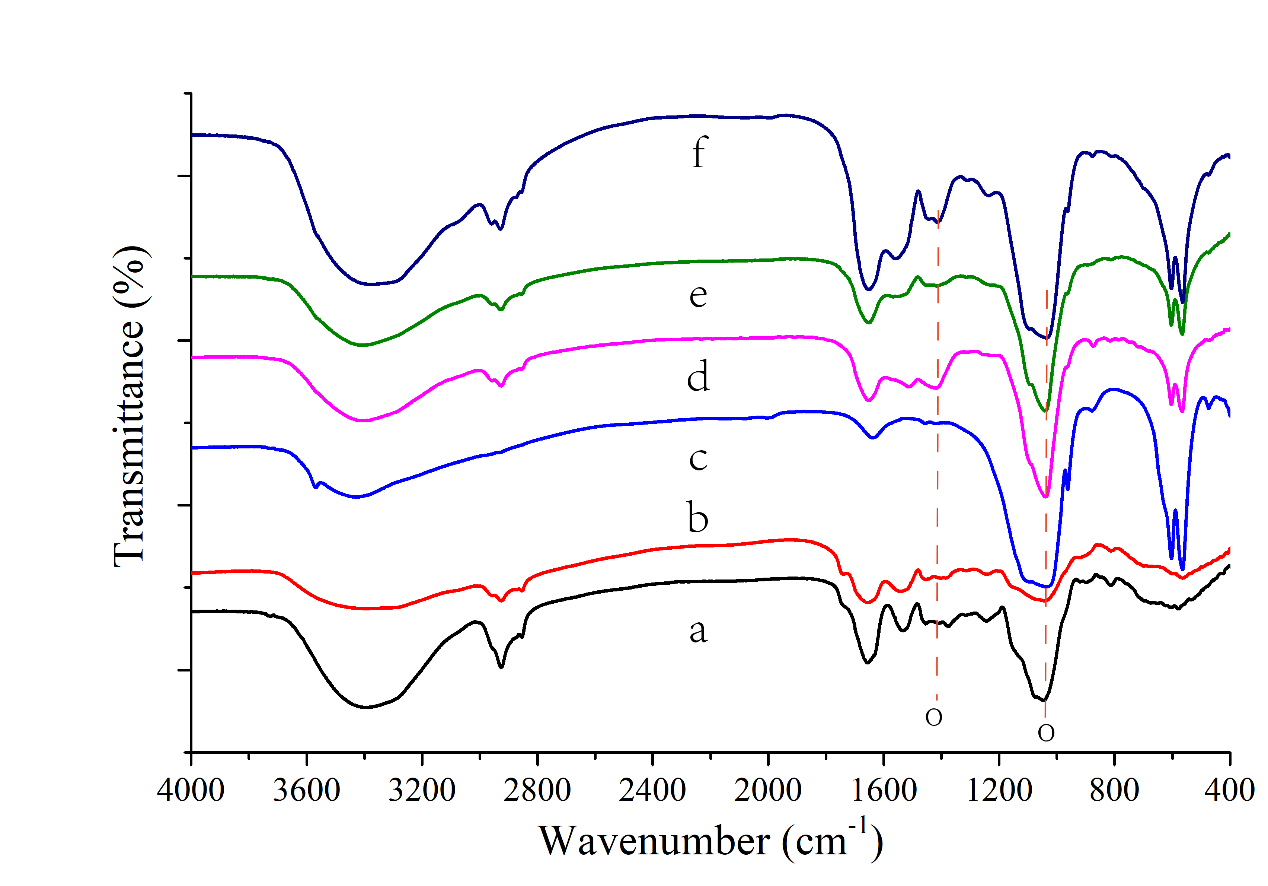


Figure S2. FTIR of minerals synthesized by S. cerevisiae in different conditions. Group4, CaCl_2_ in B4 medium; Group5, CaCl_2_ in B4 medium with urea; Group7, acetate in Czapek-Dox medium; Group9, lactate in B4 medium without glucose; Group10, CaCl_2_ in B4 medium without glucose; Group13, acetate in the medium of tryptone; o, the base peak of calcium phosphate.


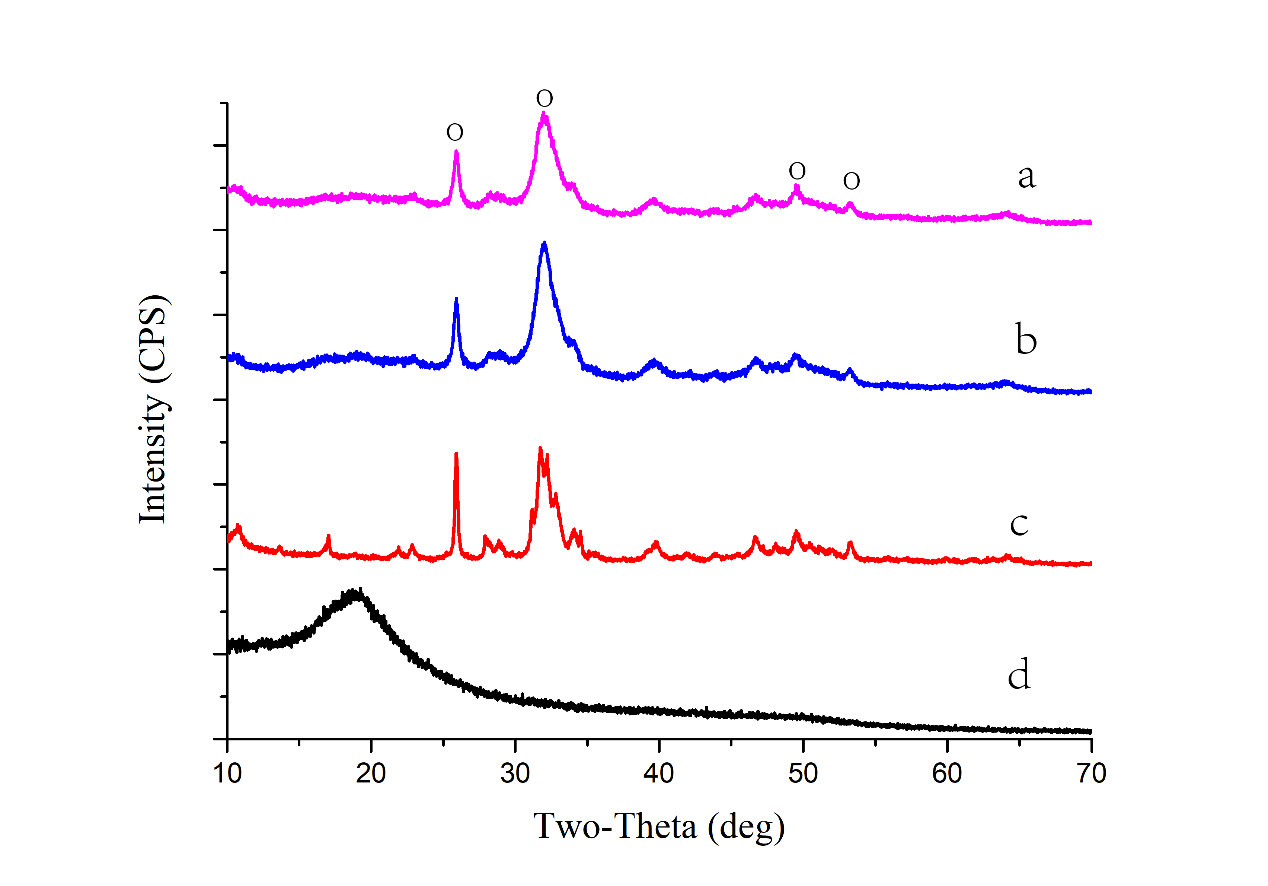


Figure S3. XRD of crystals on biomass synthesized by S. cerevisiae in different conditions. Group7, acetate in Czapek-Dox medium; Group9, lactate in B4 medium without glucose; Group10, CaCl_2_ in B4 medium without glucose; Other Groups, except the mentioned groups, the XRD results of the Groups were the same as shown in the figure; o, the base peak of calcium phosphate.


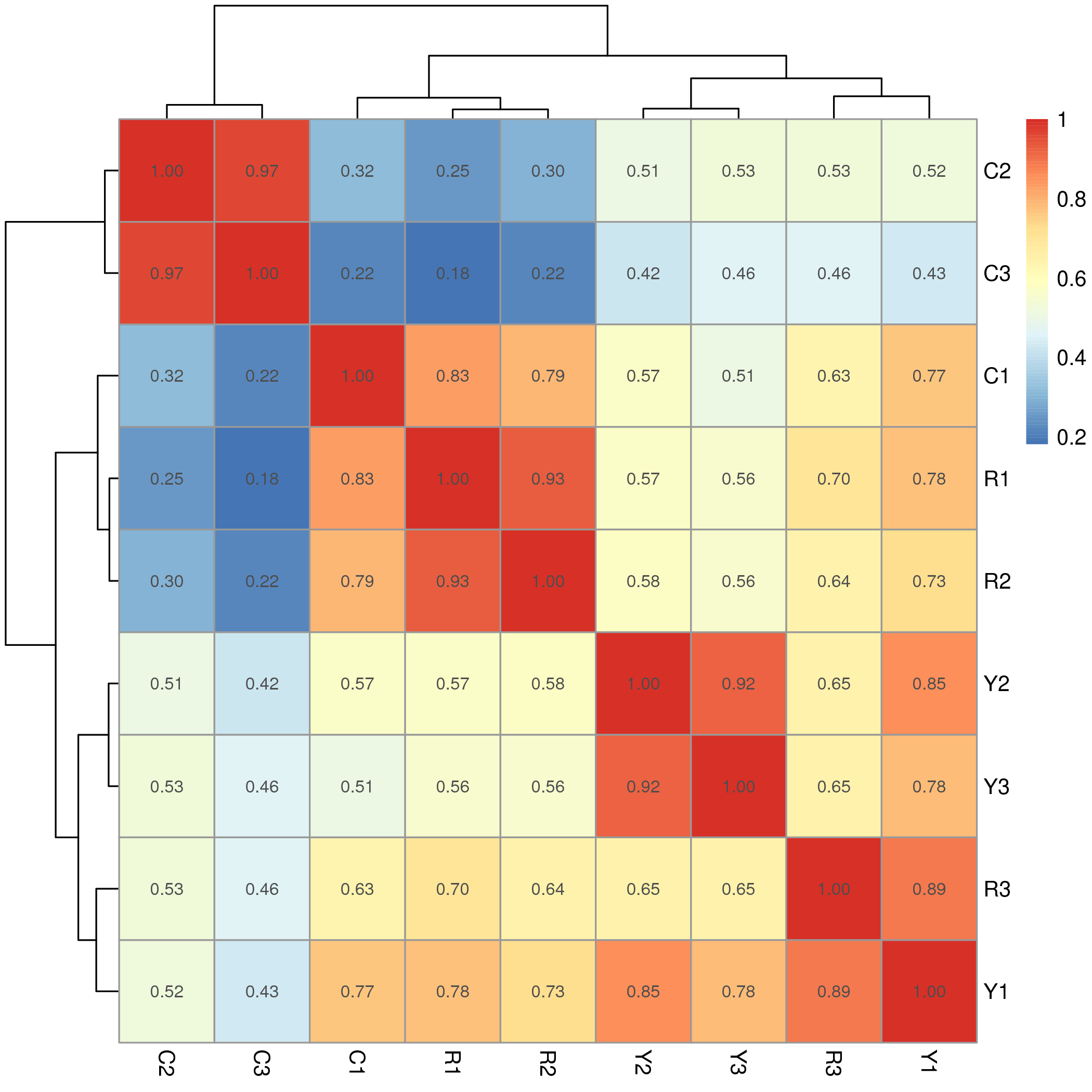


Figure S4. Pearson correlation heatmap between different samples. C1, C2 and C3, parallel samples of transcripts in response to CaCl_2_; Y1, Y2 and Y3, parallel samples of transcripts in response to acetate; R1, R2 and R3, parallel samples of transcripts in response to lactate.


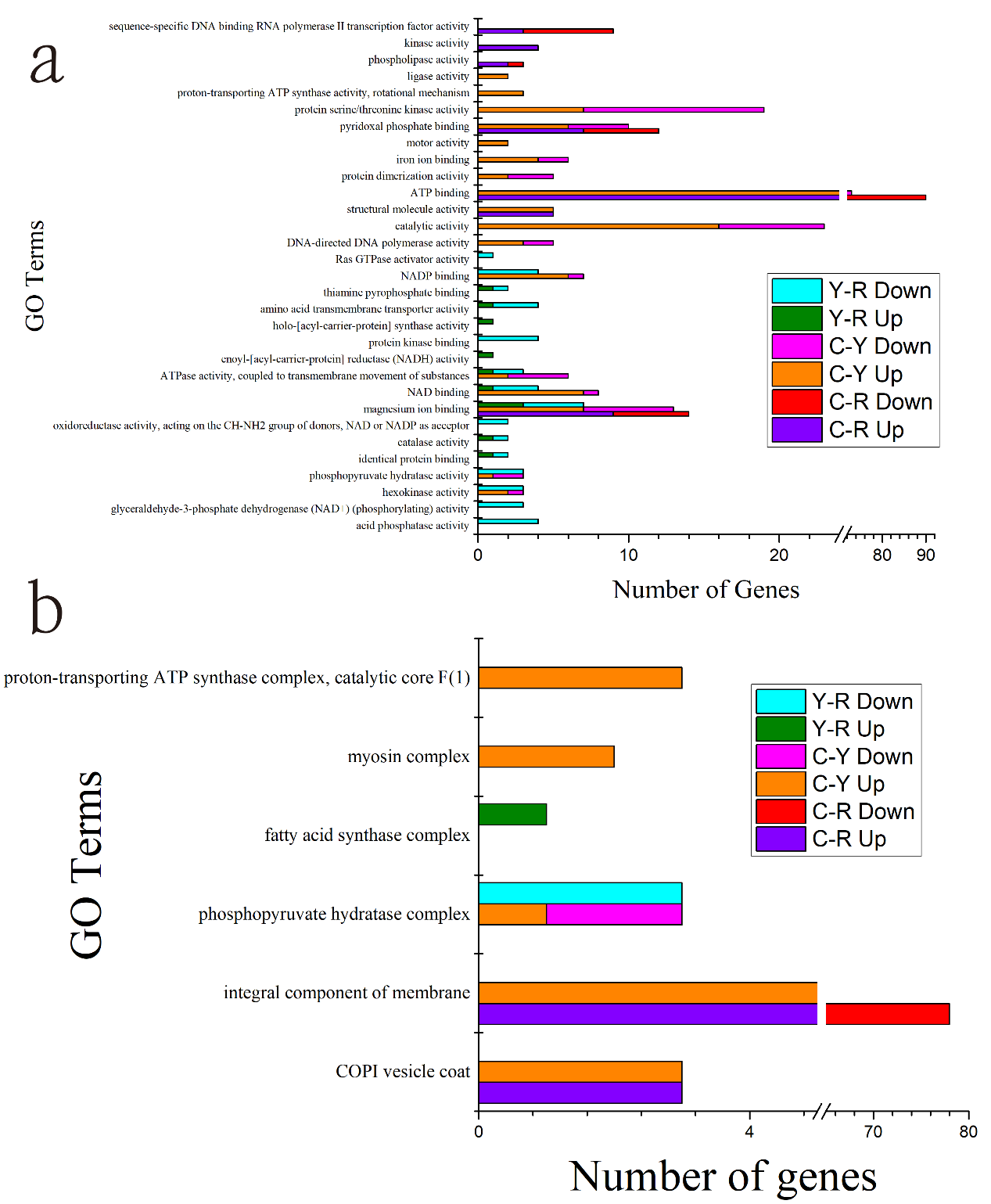


Figure S5. GO Terms of molecular function (a) and cellular components (b) identified in S. *cerevisiae* transcripts in response to different conditions. C-R, comparison of transcripts from the yeast cultured under between calcium acetate medium and control; C-Y, comparison of transcripts from the yeast cultured under between calcium lactate medium and control Y-R, comparison of transcripts from the yeast cultured under between calcium acetate medium and calcium lactate medium; Up, up-regulated DEGs; Down, down-regulated DEGs.
